# Prevalence of a dual diagnosis of mental illness and substance use disorder in Ontario, Canada: a retrospective cohort study using linked administrative data

**DOI:** 10.23889/ijpds.v9i5.2837

**Published:** 2024-09-18

**Authors:** Jesse Young, Bryan Tanner, Matthew Crocker, Isobel Sharpe, Hong Lu, Paul Kurdyak

**Affiliations:** 1 Institute for Mental Health Policy Research, Centre for Addiction and Mental Health; 2 Dalla Lana School of Public Health, University of Toronto; 3 Melbourne School of Population and Global Health, The University of Melbourne; 4 Centre for Adolescent Health, Murdoch Children’s Research Institute; 5 School of Population and Global Health, The University of Western Australia; 6 Curtin School of Population Health, Curtin University; 7 ICES; 8 Temerty Faculty of Medicine, University of Toronto; 9 Institute for Health Policy, Management, and Evaluation, University of Toronto

## Objectives

Population-based prevalence estimates of co-occurring mental illness and substance use disorder (herein dual diagnosis) are scarce and derived from a single source (e.g., survey data) which may lead to underestimation of prevalence. We linked administrative data to a representative mental health survey in Ontario, Canada to estimate dual diagnosis prevalence using a data triangulation method.

## Approach

We retrospectively linked the 2002 and 2012 Canadian Community Health Survey on Mental Health (CCHS-MH) to emergency department, inpatient hospital, and outpatient physician records in Ontario, Canada. Mental illness and substance use disorder were ascertained though self-report, Composite International Diagnostic Interview screening, and linked administrative health records. We estimated 1-year, 5-year, and lifetime prevalence of dual diagnosis.

## Results

Of the CCHS-MH survey participants, 14,790 (99.8%) were included in the study. The 1-year, 5-year, and lifetime prevalence of dual diagnosis was 2.3%, 4.8%, and 9.8%, respectively, which attenuated to 2.1%, 4.1%, and 8.4%, respectively, when tobacco use disorder was removed from substance use disorder ascertainment.

## Conclusion

Dual diagnosis is more common in the general population than previously estimated. Considering barriers to accessing care for both mental illness and substance use disorders among people with dual diagnosis annually, our 5-year prevalence estimate is likely most informative for health system planning.

## Implications

In the context of increased health burden, barriers to care access, and challenges to effective treatment associated with dual diagnosis, our findings present a case for increased investment in integrated models of mental healthcare and addiction medicine.

